# Thermal Treatment Effects on Structure and Mechanical Properties of Polybutylene Terephthalate and Epoxy Resin Composites Reinforced with Glass Fiber

**DOI:** 10.3390/polym16162269

**Published:** 2024-08-10

**Authors:** Jiangang Deng, Zhenbo Lan, Zhuolin Xu, Wei Long, Qiang Sun, Yu Nie

**Affiliations:** 1 Wuhan Nari Limited Liability Company of State Grid Electric Power Research Institute, Wuhan 430206, China; 2 State Grid Electric Power Research Institute, Nanjing 211106, China

**Keywords:** GF/PBT, GF/ER, glass fiber, thermal aging, mechanical properties, failure mechanism

## Abstract

In this study, two types of composites, polybutylene terephthalate (PBT) and epoxy resin (ER), reinforced with 20% of glass fiber (GF) are used as the comparative research objects. Their mechanical properties after thermal aging at 85~145 °C are evaluated by tensile strength and fracture morphology analysis. The results show that the composites have similar aging laws. The tensile strength of GF/PBT and GF/ER decrease gradually with the increase of aging temperature, while their elastic moduli are independent of the thermal treatment temperature. Scanning electron microscopy study of the fracture surface shows that separation of glass fiber from PBT and ER matrix becomes more obvious at higher aging temperature. The fibers on the matrix surface appear clear and smooth, and the whole pulled out GFs can be observed. As a main mechanical strength degradation mechanism, the deterioration of interface adhesion between the matrix and GF is discussed. A large difference in coefficients of thermal expansion of the matrix and GF is a main factor of the mechanical degradation.

## 1. Introduction

Polybutylene terephthalate (PBT) possesses good mechanical and electrical insulation properties and has high chemical stability [1,2,3]. It is considered to be one of the most tenacious crystalline thermoplastic engineering plastics and is widely used in automobile, ship, energy-saving lamps, outdoor communication equipment and other fields [4,5]. The epoxy resin (ER) is another thermosetting polymer with interesting set of properties (stability, adhesion, cure temperature, strength) [6]. This makes ER widely used in microelectronics, aerospace, military and other fields. Heat and oxidation by environmental oxygen typically affect material properties simultaneously. Polymers, which are easy to be oxidized, under such conditions degrade and have relatively low heat resistance [7]. The characteristics of PBT are its hydrolysis and instability at high temperature, which result in fracturing of macromolecular chain structure, reduction of PBT toughness, and narrowing of the processing window to 250–260 °C [8,9,10,11,12]. The ER also can turn yellow, crack and age with the increase of service time, the mechanical properties can deteriorate, and the service life is shortened [13,14,15,16,17,18]. To improve stability of the PBT material, functional fillers such as low-density polyethylene, polycarbonate, mixture of Fe/FeO, carbon nanotubes with relatively low loading are added [19,20,21,22,23,24]. The high temperature degradation problem of the ER can be solved by adding fiber fillers during the curing, such as glass fibers (GFs), mineral wool, carbon fibers etc. In addition, reinforcement with the fiber fillers results in improvement of mechanical properties and corrosion resistance [25,26,27,28,29,30]. Nowadays, many polymers are used in 5G mobile communication equipment components, such as transmitter circuits, which require high temperature stability. Therefore, there is an increasing need in polymer materials with good mechanical properties and stable at high temperatures. In this study, to increase mechanical performance of commercial PBT and ER polymers we reinforce them with GF. Next, we perform a thermal aging of the GF/ER and GF/PBT composites to investigate its impact on their structure, morphology, and mechanical properties. Mechanisms responsible for degradation of the mechanical properties are discussed.

## 2. Materials and Methodology

### 2.1. Materials and Sample Preparation

The composites used in this study are polybutylene terephthalate (PBT) and epoxy resin (ER) matrices reinforced with 20% GFs. PBT matrix (powder), diglycidyl ether of bisphenol A ER matrix, and 3,3-diaminodiphenyl sulfone curing agent were purchased from Honghe Co., Ltd. (Zigong, China). The GF (FR5301B-2000) with the average diameter of 10 μm was purchased from Chongqing Polymer Composite International Co., Ltd. (Chongqing, China). The fiber-reinforced GF/PBT composites were prepared by adding of GFs into melted and heated up to 240 °C PBT matrix and subsequent mixing. Preparation of the GF/ER composites included a few steps. First, mixture of ER and curing agent was prepared. Then, a layer of GF was prepared and immersed into the still liquid ER with the curing agent. After filling with the ER the GF layer was taken out and placed on a mold for subsequent curing. This operation was repeated a few times to obtain multilayer composite with a laminar structure. Dog-bone-shaped samples for mechanical tests and structural study were prepared from GF/PBT and GF/ER composites.

### 2.2. Characterization Techniques

High temperature aging treatment of the GF/PBT and GF/ER composites was performed using HZ-2004 furnace oven (Dongguan Lixian Instrument Scientific Co., Ltd., Dongguan, China) at temperatures of 85, 100, 115, 130 and 145 °C for 180 h in air atmosphere. The thermal aging treatment was performed using GB/T 2423.3-2016 standard [31]. The selected temperature range is within the region where changes in the mechanical properties and structure of the GF/PBT and GF/ER composites are observed due to heat treatment. Each sample was named as GF/PBT-x and GF/ER-x, where the x represents the aging temperature experienced and x = 0 means the sample before treatment. To study high temperature effects on mechanical properties of composites tensile test was carried out. The tensile test of dog-bone-shaped samples was conducted on a mechanical testing machine MTS E45 (MTS, Eden Prairie, MN, USA) at room temperature. The average value of the tensile properties of each sample was obtained after five measurements to reduce the experimental uncertainty. The tensile properties were evaluated using ASTM D 3039 standard [32]. The sectional fracture morphology of the samples after tensile tests were analyzed by a scanning electron microscope TESCAN MIRA III (SEM, Brno, Czech Republic). More experimental details can be found in our previous study [33].

## 3. Results and Discussion

### 3.1. Tensile Properties

**Figure 1:**
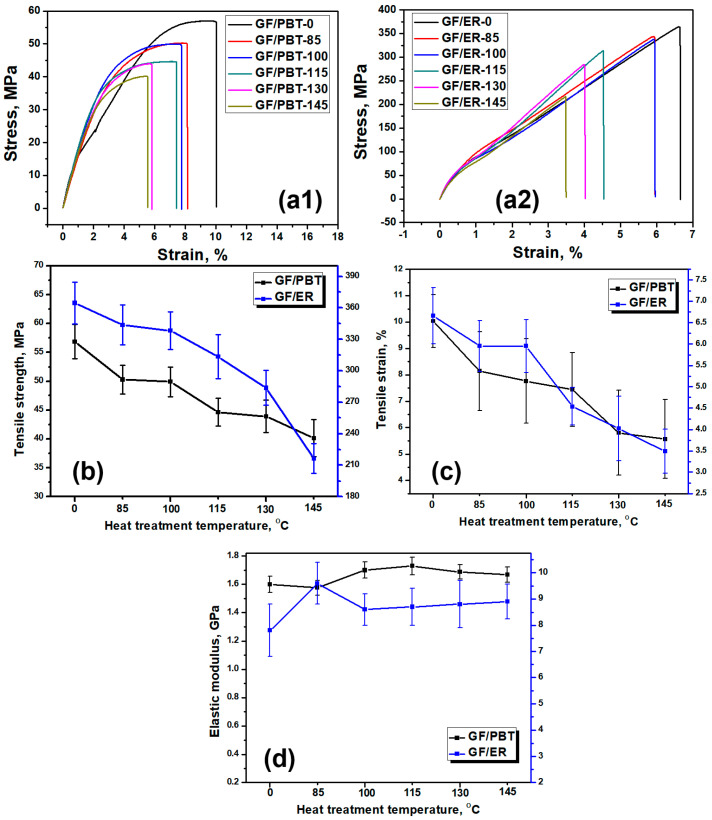
GF/PBT and GF/ER composites tensile stress-strain curves measured after heat treatment at different temperatures (**a1**,**a2**), tensile strength (**b**), GF/PBT and GF/ER composites tensile strain (**c**), and elastic modulus (**d**) measured after heat treatment at different temperatures.

The tensile stress-strain curves of the GF/PBT and GF/ER composites before and after heat treatment at different temperature are shown in Figure 1(a1) and Figure 1(a2), respectively. The stress-strain curves have two regions: a linear region and a region with nonlinear behaviour. In Figure 1(a1), the reference sample GF/PBT-0 shows nearly linear dependence on strain up to 5%. At the strain more than 5% the stress does not follow the Hooke’s law demonstrating large plastic deformation area and at a strain of 10% the sample is fractured. After heat treatment all GF/PBT demonstrates slight increase in the average curve slope within the strain region of 0–3%. However, the plastic deformation is observed earlier, which is probable due to irreversible change of the molecular structure of the PBT matrix, resulting in its lower tensile strength. Unlike the GF/PBT samples, the GF/ER samples after heat treatment do not demonstrate change in shape of the stress-strain dependence. Within all strain region the dependence nearly follows the Hooke’s law of elastic deformation without any plastic deformation. However, the heat treatment reduces both the tensile strength and strain. Compared with GF/PBT, the GF/ER composites exhibit linear stress-strain curves in all studied strain range, indicating that the elastic stress limit is higher than the tensile strength. Figure 1b, Figure 1c and Figure 1d show the tensile strength, tensile strain before sample fracture, and elastic modulus calculated at low strain, respectively, of the two composites before and after heat treatment. The tensile strength and tensile strain of both composites demonstrate decrease with the increase of heat treatment temperature. The tensile strength of GF/PBT composite before treatment is 6 times lower than that of GF/ER, with values of 57 MPa and 364 MPa, respectively. With increasing treatment temperature, the tensile strength of GF/PBT and GF/ER composites gradually decreases by 30%, from 57 to 40 MPa and by 41%, from 364 to 216 MPa, respectively. The GF/ER composite demonstrates faster degradation especially at the highest temperature of 145 °C (Figure 1b), indicating structural degradation acceleration. Similarly, the tensile strain value of GF/PBT sample decreased from 10.0% to 5.6%, and the tensile strain of GF/ER sample decreased from 6.7% to 3.5%, as shown in Figure 1c. The values of tensile strength of the GF/PBT and GF/ER composites before treatment, 57 and 364 MPa, are in agreement with literature data on fiber reinforced composites 70–80 MPa [34,35] and 230–306 MPa [36,37], respectively. Different geometric parameters of GFs can explain differences in tensile strength data for each material. In Figure 1d the elastic modulus of two composites as function of treatment temperature are shown. Within the range of experimental uncertainty, the elastic modulus does not demonstrate any change with temperature, which can be explained by the fact that the selected definition of elastic modulus is related to the slope tangent of the stress-strain curve in the low strain region. Whereas, significant changes in stress-strain dependence are observed at larger strains.

The obtained data shows that thermal aging treatment leads to the ductile degradation of the GF/ER and GF/PBT composites. The mechanical properties of GF/PBT and GF/ER composites significantly depend on interaction between GF and matrix. Lal et al. have shown that debonding of the matrix from the GF surface is one of the most characteristic failure modes of GF reinforced composites [38]. Therefore, the reduction of interfacial adhesion between GF and matrix after heat treatment is one of the reasons for the mechanical properties’ degradation, see below. Oxidation of the PBT and ER matrices during the heat treatment as well as difference in coefficients of thermal expansion (CTEs) of the matrices and GF can be reasons of interfacial adhesion degradation. Moreover, effects of mechanical properties degradation are also related to the failure of the internal structures of the composite matrices. During thermal aging, PBT and ER are prone to cross-linking and fracture of molecular structure, and increase of crystallinity of materials due to oxidation. The change of molecular structure and crystallinity is a major reason for increasing the brittleness of materials.

### 3.2. Microscopic Analysis of Fracture Morphology

**Figure 2:**
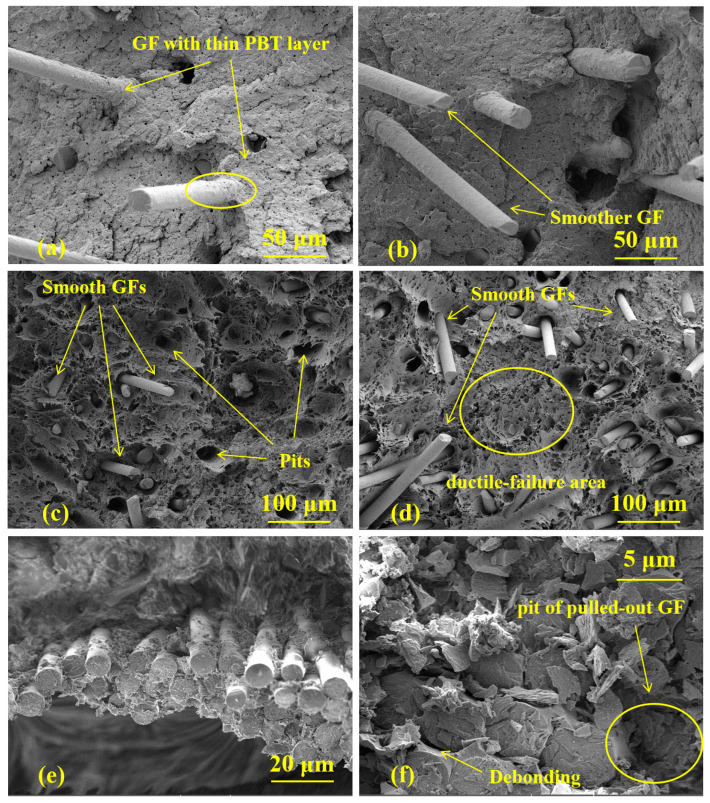
Fracture morphology after aging at different temperatures: samples GF/PBT-0 (**a**), GF/PBT-115 (**b**), GF/PBT-130 (**c**), GF/PBT-145 (**d**), GF/ER-0 (**e**), and GF/ER-115 (**f**) taken at a higher magnification.

Figure 2 shows the SEM fracture section images of GF/PBT and GF/ER composites before and after heat treatment at different temperatures at a higher magnification. Before the heat treatment well embedded into PBT and ER matrices GFs are well observed, Figure 2a,e. GFs surface of the not treated GF/PBT composite is well covered with matrix suggesting a good adherence of two materials, Figure 2a. After the heat treatment of the GF/PBT composite at 115 °C the sample shows that pulled out GFs surface becomes smoother, Figure 2b, which means beginning of degradation of the adherence with PBT matrix. Therefore, it can be concluded that GF/PBT composite can withstand temperature treatment up to 100 °C. After the heat treatment at 130 and 145 °C the samples demonstrate multi-fracture modes: debonding of the GF from the matrix, breakage of the GFs, and GF pulling-out, Figure 2c,d. Moreover, at these temperatures the adhesion of the GF to the matrix is significantly reduced, the root of the glass fiber and the PBT matrix are not tightly adhered, and gradually separated from each other. In addition, a large number of pores can be observed on the fracture surface, especially at higher temperature, which means that the matrix fractured in a ductile-failure mode [39].

The SEM image of the heat-treated GF/ER-115 sample is shown in Figure 2e. After the heat treatment slits between GFs and ER matrix are observed, suggesting beginning of adherence degradation between GF and ER matrix. After heat treatment temperature is further increased to 130 and 145 °C, the separation between GF and matrix is more obvious. Compared with the GF/PBT composite, the GF/ER composite has less serious interfacial degradation. Therefore, it can be concluded that after heat treatment at temperatures higher than 115 °C the polymer–fiber interface is weaker than the polymer strength, and weakening of the polymer–fiber interface correlates with increase of temperature.

The observed interfacial degradation after high temperature heat treatment should be related to the thermal stress at the matrix-fiber interface due to difference in CTEs of PBT and ER matrices and GFs. The CTEs of not reinforced PBT and ER matrices are 14–15·10^−5^ K^−1^ [40] and 7–9·10^−5^ K^−1^ [41], respectively. For the CTE of the GFs we can use CTE of the α-quartz, which is in the range of 7.1–13.1·10^−6^ K^−1^ depending on crystal orientation [42]. Therefore, the CTEs of the matrices are one order of magnitude higher than that of the GF and during the heat treatment such difference in CTEs should inevitably lead to generation of stress at the matrix-fiber interface, which results in interface damage, fiber smoothness, fiber shedding and other phenomena. The debonding of matrix from the GF after heat treatment initiates the process of defect formation on the interface surface. In the followed tensile experiments these defects become responsible for brittle fracture of the matrix. At a higher heat treatment temperature more interface defects are formed, therefore, brittle fracture is observed at a lower stress and strain value. The less serious interfacial degradation of GF/ER composite observed in SEM images can be related to the lower CTE of the ER matrix as compared to that of the PBT matrix.

## 4. Conclusions

In this paper, polybutylene terephthalate (PBT) and diglycidyl ether of bisphenol A (ER) reinforced with 20% of glass fiber (GF) have been studied to compare the effect of heat treatment in the temperature range from 85 to 145 °C on tensile properties. The GF/PBT composites were prepared as homogeneous mixture of PBT matrix and GF. The GF/ER was a multilayer composite with laminar structure. With increasing treatment temperature, the tensile strength of GF/PBT and GF/ER composites gradually decreases from 57 to 40 MPa and from 364 to 216 MPa, respectively, which is 29 and 41% of initial values, respectively. Within all strain region the stress-strain dependence of the GF/ER composite follows the Hooke’s law of elastic deformation and the GF/PBT composite demonstrates large plastic deformation area before the failure. Scanning electron microscopy (SEM) study of the fracture morphology demonstrates that the GF/PBT composites has a more serious interface degradation after heat treatment than that of the GF/ER. The heat-treated GF/PBT fracture surface does not show obvious crack initiation and propagation signs. At higher aging temperatures (130 and 145 °C), ductile-failure mode is observed. SEM studies of the heat-treated GF/PBT and GF/ER composites reveal the GFs with smooth surface appeared on the fracture surface, indicating reduction of the adherence between PBT and ER matrices and GFs. Degradation of the adhesion we explain by the large difference in the values of the coefficients of thermal expansion of matrices and GF, which is one order of magnitude. Failure of the interface between PBT and ER matrices and GF results in reduction of the “reinforced” part of the tensile strength. This work is helpful to understand the effects of thermal aging on mechanical performance of the GF/PBT and GF/ER composites. The composites can be used in mobile communication transmitter circuits where high temperature stability of the components is required.

## Data Availability

The original contributions presented in the study are included in the article, further inquiries can be directed to the corresponding authors.
